# UWV-Yolox: A Deep Learning Model for Underwater Video Object Detection

**DOI:** 10.3390/s23104859

**Published:** 2023-05-18

**Authors:** Haixia Pan, Jiahua Lan, Hongqiang Wang, Yanan Li, Meng Zhang, Mojie Ma, Dongdong Zhang, Xiaoran Zhao

**Affiliations:** School of Software, Beihang University, Beijing 100191, Chinazhaoxiaoran@buaa.edu.cn (X.Z.)

**Keywords:** underwater video, object detection, coordinate attention, loss function, frame-level optimization

## Abstract

Underwater video object detection is a challenging task due to the poor quality of underwater videos, including blurriness and low contrast. In recent years, Yolo series models have been widely applied to underwater video object detection. However, these models perform poorly for blurry and low-contrast underwater videos. Additionally, they fail to account for the contextual relationships between the frame-level results. To address these challenges, we propose a video object detection model named UWV-Yolox. First, the Contrast Limited Adaptive Histogram Equalization method is used to augment the underwater videos. Then, a new CSP_CA module is proposed by adding Coordinate Attention to the backbone of the model to augment the representations of objects of interest. Next, a new loss function is proposed, including regression and jitter loss. Finally, a frame-level optimization module is proposed to optimize the detection results by utilizing the relationship between neighboring frames in videos, improving the video detection performance. To evaluate the performance of our model, We construct experiments on the UVODD dataset built in the paper, and select mAP@0.5 as the evaluation metric. The mAP@0.5 of the UWV-Yolox model reaches 89.0%, which is 3.2% better than the original Yolox model. Furthermore, compared with other object detection models, the UWV-Yolox model has more stable predictions for objects, and our improvements can be flexibly applied to other models.

## 1. Introduction

Underwater video object detection refers to recognizing objects in videos. With the rapid development of deep learning, more and more researchers are applying deep learning to solve the problem of object detection, effectively improving the accuracy and robustness [1].

In underwater environments, images are often blurry and the contrast is reduced due to the absorption, reflection, and refraction of light, which ultimately reduces the accuracy of detection. To address these challenges, one approach is to indirectly improve detection performance by enhancing the quality of underwater images [2,3,4,5,6,7]. Another approach used to optimize the detection performance is to improve the object detection model [8,9,10].

Compared to image-level object detection, video-level object detection contains richer temporal and spatial information. Common video object detection methods can be divided into four categories [11]. (1) The first is to convert video object detection into image object detection. The spatial information from the videos is then used to optimize the detection results, which is called post-processing, such as the Seq-NMS method [12]. (2) The second is to integrate the spatial and temporal information into the video object detection network and learn relevant information during model training [13,14]. (3) The third is based on feature filtering, where the model focuses on the relative key features and suppresses or discards unnecessary calculations [15]. (4) The fourth is to use efficient neural networks for video object detection [16], improving the detection accuracy and speed through special network designs.

Most existing research on object detection focuses on non-underwater scenes or underwater image object detection. However, existing object detection models have limited detection capabilities for underwater videos with blurry images and low contrast. For objects with blur and low contrast, it is difficult for the model to obtain their detailed features, so they are often undetected or detected as other categories, which reduces the accuracy and recall of detection. In addition, they cannot also utilize the relationships between adjacent frames in the videos. The detection results of the same object between adjacent frames often have large differences in confidence and experience a serious jitter phenomenon, which affects the overall effect of video detection. As a result, their detection performance is unsatisfactory for underwater videos. Therefore, we propose an improved Yolox video object detection model named UWV-Yolox.

Considering the low contrast of the underwater video, we believe that enhancing the contrast of the input is conducive to recovering the feature of the object, so we first use the Contrast Limited Adaptive Histogram Equalization method to enhance the contrast of the input. Second, our intuition is that augmenting the representations of the objects of interest in the model is beneficial for learning more feature information about the object. Therefore, we integrate the coordinate attention module [17] into the backbone module to propose the CSP_CA module, which integrates the location information into the feature map. The loss function is used to evaluate the gap between the predicted object and the ground truth object. In order to reduce the gap, we propose a new jitter loss function and $CIOU_{log}$ regression loss. The jitter loss utilizes the change in object position acceleration of the adjacent frames in the video to suppress jitters. The $CIOU_{log}$ regression loss uses overlapping area, position, and shape into consideration to make the predicted objects similar to the ground truth objects. Finally, for the same object detected in the video, we consider that good detection results can optimize the poor results. Thus, we propose a frame-level optimization module, which links tubelets for re-scoring and re-coordinating.

Our main contributions are as follows:

1. We use the Contrast Limited Adaptive Histogram Equalization method to enhance the contrast of underwater videos, making the model more suitable for underwater detection. We also propose a new CSP_CA module by integrating Coordinate Attention to augment the representations of the objects of interest;
2. We propose a new loss function, which includes classification loss, regression loss, confidence loss, and jitter loss, to improve the model’s performance in video detection;
3. We propose a frame-level optimization module, which uses tubelet linking, re-scoring, and re-coordinating to optimize the model’s results in video object detection.

The rest of this paper is organized as follows. Section 2 shows the research related to underwater video object detection. Section 3 introduces the methods of the proposed UWV-Yolox model. Section 4 presents the experiments conducted on the UVODD dataset built in this paper to validate the effectiveness of the proposed underwater object detection model. Section 5 provides the conclusion of this paper.

## 2. Related Work

### 2.1. Image-Level Underwater Object Detection

One way to improve detection performance is to enhance the quality of the underwater images. Karel et al. proposed Adaptive Histogram Equalization (AHE) and Contrast Limited Adaptive Histogram Equalization (CLAHE) for contrast enhancement, respectively [2]. In 2007, Kashif et al. proposed a sliding stretch-based method to enhance images [3]. In 2018, Huang et al. proposed a relative global histogram stretching method based on adaptive parameter acquisition to correct the contrast and color of images [4].

The above are traditional image augmentation methods. In recent years, deep learning has been applied to image augmentation. Li et al. used Convolution Neural Network (CNN) to construct an image dehazing model in 2017, which can generate augmented images end-to-end [5]. In 2021, Fu et al. proposed a dehazing network using a 2D discrete wavelet transform to enhance images [6]. In 2022, Liu et al. proposed a Cycle Generative Adversarial Network (Cycle-GAN), which achieved image dehazing under unsupervised training [7].

Another way to optimize the detection performance is to improve the object detection model. In 2021, Zhang et al. proposed a Yolov4-based lightweight underwater object detection method and multi-scale attentional feature fusion module to trade off accuracy and speed [8]. In the same year, Zhang et al. fused Yolov4 and FaceNet models for feature extraction and prediction to improve the recognition of fish underwater [9]. In 2022, Li et al. proposed an improved Ghost-Yolov5 network based on an attention mechanism, using Ghostconvolution from ChostNet to replace the convolution kernels in Yolov5 and adding an attention mechanism to the feature extraction network [10].

These image-level object detection methods have improved detection accuracy, but they only consider the information from single images. For video object detection, there is still rich spatiotemporal information that has not been fully utilized.

### 2.2. Video-Level Object Detection

In 2016, Kang et al. proposed a complete framework for the VID task based on image-based object detection and object tracking. They utilized a temporal convolution network to optimize the detection results [18]. In 2021, He et al. proposed an end-to-end video object detection model based on a spatiotemporal transformer architecture, presenting a temporal transformer to aggregate both the spatial object queries and the feature memories of each frame [19]. Zhao et al. proposed the first weakly supervised video salient object detection network based on fixed-guided scribble annotation, effectively fusing visual dynamic features through the visual dynamic fusion module and velocity information enhancement module [20].

These video object detection methods have achieved great results in conventional detection tasks, but in underwater scenes, they still cannot reach a reasonable detection result.

## 3. Methods

**Figure 1:**
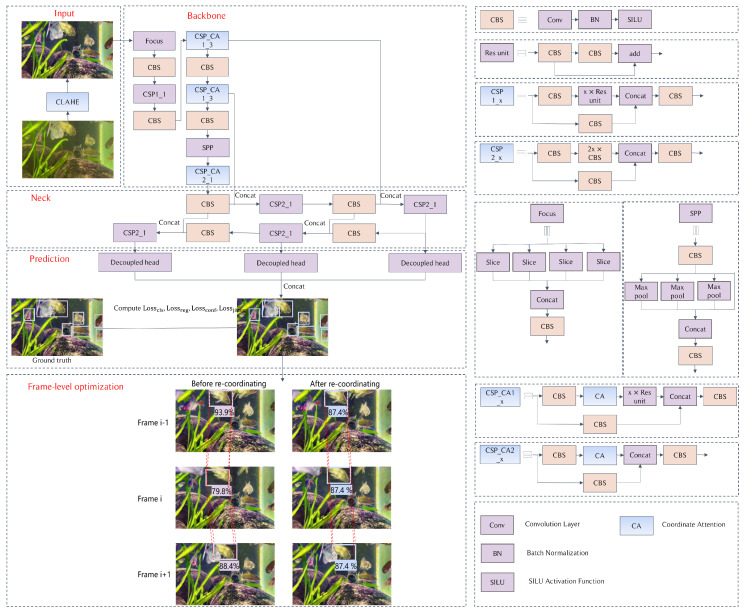
UWV-Yolox structure.

The structure of the UWV-Yolox model is shown in Figure 1. It can be divided into five modules: input, backbone, neck, prediction, and a frame-level optimization module. In the input module, we add Contrast Limited Adaptive Histogram Equalization to enhance the input videos. We propose the CSP_CA module to replace the CSP module in the backbone module by integrating a Coordinate Attention module. In the prediction module, we propose jitter loss and regression loss. Finally, we add a frame-level optimization module to optimize the detection results.

### 3.1. Video Contrast Enhancement

In underwater environments, the contrast of the underwater videos is usually low, which causes interference with the feature information of the objects. To solve this problem, we recover more feature information in the underwater video by enhancing the contrast to improve the detection effect. Contrast Limited Adaptive Histogram Equalization (CLAHE) is an adaptive histogram equalization method, which divides the input into small blocks and then performs limited histogram equalization. Blocking and limiting contrast before equalization can avoid overexposed areas and suppress noise. Therefore, we use the CLAHE method to enhance the contrast of the underwater video.

Contrast Limited Adaptive Histogram Equalization (CLAHE) improves Histogram Equalization (HE). For a grayscale image {x}, let $n_{g}$ be the number of times the grayscale *g* appears. Then the probability of grayscale *g* appearing in the entire grayscale image is defined as follows:

(1) $$p_{x}\left(g\right)=p\left(x=g\right)=\frac{n_{g}}{n},0\leq g<L$$

The total number of gray values is denoted by *L* (usually 256), and n represents the total number of pixels in the image. Therefore, the cumulative distribution function of the gray level can be expressed as Equation (2), which is also the cumulative normalized histogram of the gray image.

(2) $$f_{x}\left(g\right)=\sum_{t=0}^{g}p_{x}\left(x=t\right)$$

Then, through the transformation of y=T(x), the original gray-scale image {x} is transformed into the contrast-enhanced gray-scale image {y}. The specific linear cumulative distribution function of the enhanced image is defined as follows:

(3) $$f_{y}\left(g\right)=kg$$

The relationship between the cumulative distribution function of the original gray image and the enhanced image is shown in Equation (4), and *a* is a constant.

(4) $$f_{y}\left(y^{\prime }\right)=f_{y}\left(T(a)\right)=f_{x}\left(a\right)\;,\;0\leq a<L$$

The function T(·) maps *a* to the range of [0,1] using the normalized histogram. To remap the values back to the original range, a transformation needs to be applied to the results to obtain the final enhanced gray-level image {y}, as shown in Equation (5).

(5) $$y^{\prime }=y\left(\max\left\{f_{v}\right\}-\min\left\{f_{v}\right\}\right)+\min\left\{f_{v}\right\}$$

In this way, the grayscale image {x} can be transformed into the contrast-enhanced grayscale image {y} after Histogram Equalization.

CLAHE first divides the input image into several non-overlapping blocks of equal size. It then calculates the histogram of each sub-block and computes the clipping threshold. After pixel redistribution, it performs Histogram Equalization. Finally, it reconstructs the grayscale values of the pixels. The clipping threshold prevents noise amplification in regions that are almost constant—but with noise. It limits the slope of the transformation function by clipping the histogram to a predefined value before computing $f_{y}$. In CLAHE, the clipping threshold is proportional to the slope of the transformation function, which is proportional to the slope of the cumulative distribution function $f_{x}$ in the neighborhood. Therefore, it is proportional to the value of the histogram at that pixel location. The part of the histogram that exceeds the clipping limit is not discarded but is instead evenly distributed among all histogram blocks.

**Figure 2:**
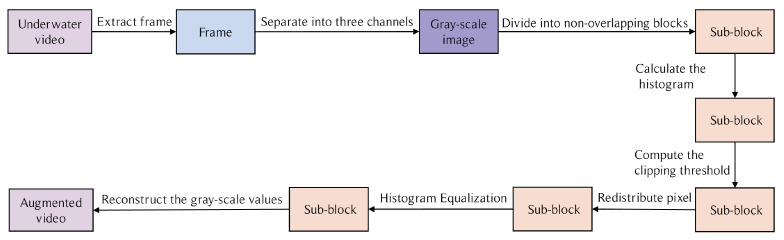
Process of the CLAHE method.

While applying CLAHE to a video, the video is first extracted into several frames, then each frame is separated into three color channels: Red, Green, and Blue (RGB). Next, CLAHE is applied separately to each color channel to enhance the contrast. Finally, we convert the augmented data back into a video with enhanced contrast. The entire process is shown in Figure 2.

### 3.2. CSP_CA Module

Blurring is inevitable in underwater videos, which makes it harder for the model to accurately detect objects. In this paper, we propose an improved CSP module named CSP_CA by integrating a Coordinate Attention (CA) module into the CSP module. It improves the detection performance on blurry underwater videos.

**Figure 3:**
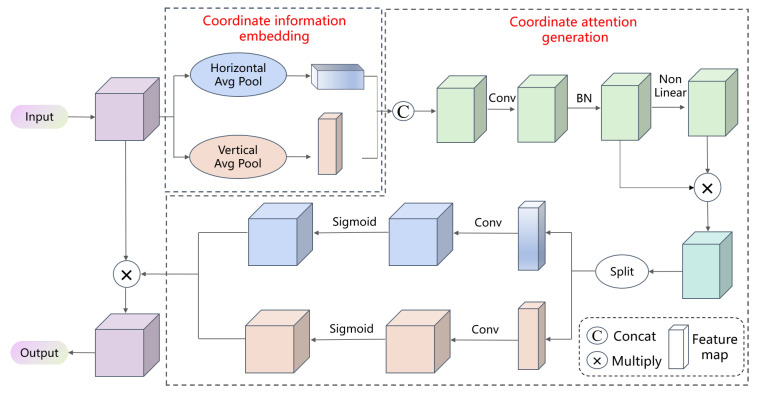
Coordinate attention module.

As shown in Figure 3, the CA module can be divided into two steps: coordinate information embedding and coordinate attention generation. Coordinate information embedding is performed first. It does not use two-dimensional global pooling to compress the feature tensor into a single feature tensor, which would cause the loss of positional information. The CA module decomposes both horizontal and vertical global pooling into one-dimensional feature encoding operations. So, it allows the model to capture long-term dependencies between channels along one direction and preserves object positional information along the other direction to help the network more accurately locate the interesting objects. Then, the CA module performs coordinate attention generation. The two cascaded feature maps are transformed using a shared 1×1 convolution, followed by normalization and nonlinear transformation to generate an intermediate feature map. Then, the intermediate feature map is split along the horizontal and vertical directions into two separate tensors. Each tensor is transformed into the same number of channels as the input feature map using a 1×1 convolution. After expansion, the two tensors are used as attention weights and added to the input feature map through multiplication to add positional information. Unlike the channel attention mechanism that only focuses on channel information, the CA module also encodes spatial information.

**Figure 4:**
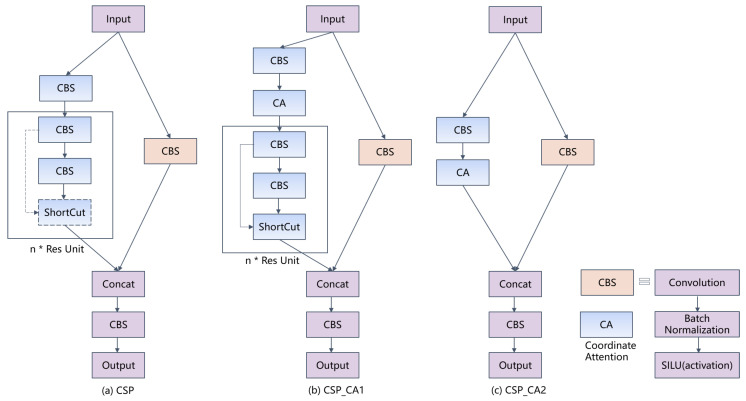
CSP_CA module and CSP module.

In the UWV-Yolox model, we propose the CSP_CA module by integrating the CA module into the CSP module. The structure of the CSP module is shown in Figure 4a. It divides the input feature map into the trunk branch and the residual branch. The residual branch directly performs convolution to extract features, while the trunk branch convolves first, and then goes through multiple Res Unit structures. Due to the different structures of the Res Unit, the CSP module includes CSP1 and CSP2 structures. The Res Unit structure in the CSP1 module adopts a residual structure. It extracts features through the residual structure of two consecutive convolutional layers. The Res Unit structure in the CSP2 module is directly composed of two consecutive convolutional layers. The two branches are then merged to output the feature map through concat operation.

There are two structures of the CSP_CA module, namely CSP_CA1 and CSP_CA2, as shown in Figure 4b,c. For the Res Unit in the original CSP that contains the residual structure, it is replaced by the CSP_CA1 module. A CA module is added between the CBS module and the Res Unit module in its trunk branch, which enhances the ability to extract blurry features. The Res Unit module in the original CSP module that does not contain the residual structure is replaced by the CSP_CA2 module. The Res Unit module is replaced by the CA module in its trunk branch, reducing the number of parameters of the model [21]. The CSP_CA module improves the detection accuracy of the UWV-Yolox model without significantly reducing the detection speed. The CSP_CA module extracts the position information along both the horizontal and vertical directions. Each element in the two attention maps reflects whether the object of interest exists in the corresponding row and column. Two-direction location is beneficial for exacting the position of the object of interest. Therefore, the CSP_CA module can augment the representations of the objects of interest and have better robustness to noise and interference in the input data, thereby improving the performance and generalization ability.

### 3.3. Loss Function

The loss function of the UWV-Yolox model includes classification loss, regression loss, confidence loss, and jitter loss, which is defined as follows:

(6) $$Loss=\lambda_{cls}Loss_{cls}+\lambda_{reg}Loss_{reg}+\lambda_{conf}Loss_{conf}+\lambda_{jitter}Loss_{jitter}$$

Both the classification loss and the confidence loss use Binary CrossEntropy (BCE) loss, as shown in Equation (7), where *y* represents the value of ground truth and p(x) represents the value of prediction. The classification loss calculates the loss of class between the prediction and ground truth, and the confidence loss calculates the loss of classified confidence.

(7) $${Loss}_{BCE}=-ylog\left(p\left(x\right)\right)+\left(1-y\right)\log\left(1-p\left(x\right)\right)$$

In object detection tasks, bounding boxes are used to locate the objects. To evaluate the quality of the predicted boxes, the closeness between the predicted box and the ground truth box is calculated, which is also known as the regression loss. The IOU loss is commonly used as regression loss. The IOU between two detection boxes A and B is defined as the intersection over the union of their areas, as shown in Equation (8). The larger the IOU, the closer the predicted box is to the ground truth box, and the smaller the loss should be. Therefore, the IOU regression loss function can be expressed as follows:

(8) $$IOU=\frac{A\cap B}{A\cup B}=\;\frac{A\cap B}{A+B-A\cap B}$$

(9) $${Loss}_{reg}=1-{IOU}^{2}$$

However, IOU only considers the relationship between the area of the predicted bounding box and the ground truth bounding box, ignoring the information about their position and shape. Therefore, only using IOU to calculate the regression loss is not accurate enough. In order to more comprehensively utilize the relationship between the predicted bounding box and the ground truth bounding box, a new regression loss function $CIOU_{log}$ is used in the UWV-Yolox model, which considers the overlapping area, position, and shape of two bounding boxes, as shown in Equation (10).

(10) $$CIOU_{log}=IOU_{log}-\left(\frac{\rho^{2}}{c^{2}}+\alpha v\right)$$

$IOU_{log}$ represents the overlap area loss, which is defined as follows:

(11) $$IOU_{log}=\frac{\log\left(w_{i}+\lambda \right)\left(h_{i}+\lambda \right)}{\log\left(w_{1}+\lambda \right)\left(h_{1}+\lambda \right)+\log\left(w_{2}+\lambda \right)\left(h_{2}+\lambda \right)-\log\left(w_{i}+\lambda \right)\left(h_{i}+\lambda \right)}$$

The size differences of the detected bounding boxes are relatively large when taking into account the different sizes of underwater objects. It makes a significant difference in the value of the IOU. In order to make the IOU loss more stable in this situation, the logarithm of the area is taken before calculating IOU. At the same time, in order to avoid the situation where the value of the area is too small so the logarithm result becomes negative and the size difference becomes large, λ is added to the bounding box’s edge length before taking the logarithm. So that it maps the values of edge length to regions where the logarithmic function changes relatively smoothly In the UWV-Yolox model, λ is set to 1 to map the values to a range greater than 0. $IOU_{log}$ also has a larger value than IOU, which increases the proportion of overlap area loss in the entire regression loss.

$\frac{\rho^{2}}{c^{2}}$ represents the position loss, where $\rho^{2}$ represents the distance between the centers of the two boxes, and $c^{2}$ represents the diagonal length of the outer rectangle of the two boxes. αv represents the shape loss, where *v* represents the difference in aspect ratio between the two boxes. It is calculated using the inverse trigonometric function, as shown in Equation (12). α is calculated by *v* and $IOU_{log}$, as shown in Equation (13).

(12) $$v=\frac{4}{\pi^{2}}{\left(arctan\frac{w_{1}}{h_{1}}-arctan\frac{w_{2}}{h_{2}}\right)}^{2}$$

(13) $$\alpha =\frac{v}{1-{IOU}_{log}+v}$$

The larger the value $IOU_{log}$ is, the closer the predicted bound box is to the ground truth bound box. Above all, the $CIOU_{log}$ regression loss function can be expressed as follows:

(14) $${Loss}_{reg}=1-{CIOU}_{log}$$

In video object detection, different frames in a video are individually detected. However, the detector’s accuracy varies for each frame, so the predicted bounding boxes of adjacent frames may vary significantly, which is not consistent with the fact that the bounding boxes of adjacent frames change in a certain pattern. This phenomenon is called jitter. It not only reduces the accuracy of video object detection but also affects the visualization of the detection results. To reduce the jitters, the UWV-Yolox model adds a new jitter loss function. The physical concepts of velocity and acceleration are incorporated to define the velocity and acceleration of the bounding boxes. Velocity is defined as the difference between the bounding box coordinates of adjacent frames, as shown in Equation (15). Acceleration is defined as the difference between the velocities of adjacent frames, which is defined as follows:

(15) $$V_{i}=Y_{i}-Y_{i-1}$$

(16) $$A_{i}=V_{i}-V_{i-1}$$

The jitters of the bounding boxes are mainly reflected in the irregular velocities of the bounding box coordinates between adjacent frames, which means that the acceleration of the bounding boxes is different. When detecting the same object in adjacent frames, the object originally changes at a certain acceleration. However, due to the predicted deviation of the object detection in one frame, the acceleration of the object changes. By suppressing the change of acceleration to reduce thhe detection errors, the jitters can be suppressed Therefore, the jitter loss mainly consists of acceleration loss, which calculates the difference between the predicted box acceleration and the ground truth box acceleration between adjacent frames, as shown in Equation (17).

(17) $${Loss}_{jitter}=\frac{1}{T-2}\sum_{i=0}^{T-2}|A_{i}^{gt}-A_{i}|$$

*T* refers to the batch size, and $A_{i}^{gt}$ represents the acceleration of the ground truth box. In the UWV-Yolox model, considering that the value of the jitter loss is heavily larger than other loss functions, the weight coefficient of the jitter loss is set to 0.05.

### 3.4. Frame-Level Optimization

**Figure 5:**
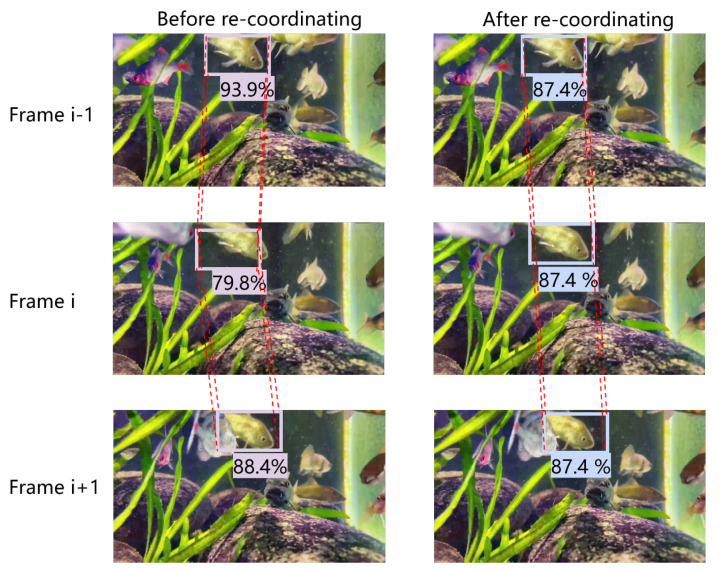
Effect of the frame-level optimization module.

In video object detection, there may be significant differences in the detection results for the same object between adjacent frames, resulting in sudden drops in confidence and bounding box jitters. Therefore, the UWV-Yolox model adds a frame-level optimization module, including tablet linking, re-scoring, and re-coordinating. The effect of the frame-level optimization module is shown in Figure 5.

The tubelet linking aims to link the detected bounding boxes of the same object across adjacent frames. The good detection results can therefore optimize the poor detection results in the same tubelet. To determine whether two detection results belong to the same object, a similarity function between the two objects needs to be defined. In this frame-level optimization module, the Intersection over Union (IOU) between two bounding boxes, the distance between the center of the bounding boxes $d_{center}$, and the aspect ratio of the bounding boxes $ratio_{w},ratio_{h}$ are chosen as evaluation metrics. Then a logistic regression function outputs a score that represents the similarity between two objects by calculating the evaluation metrics. The product of this score and the corresponding class confidences is defined as the similarity score between two objects, which is defined as follows:

(18) $$score=X\left(IOU,\;d_{center},{ratio}_{w},{ratio}_{h}\right)\times {score}_{A}\times {score}_{B}$$

X(·) refers to the logistic regression function, and ${score}_{A}$ and ${score}_{B}$ refer to the classification confidence scores of the two detected objects A and B, respectively. After determining the similarity function, a scoring matrix can be created for each pair of detection results between two frames based on their similarity scores. All paired detection objects between the two frames are then selected from the score matrix. The selection process starts by choosing the pair with the highest similarity score and then setting the corresponding rows and columns in the matrix to zero. This process is repeated until there are no more available pairs to be selected. Then, all paired detection objects between the two frames are obtained.

After obtaining all paired detection objects between two frames, the tubelet linking process begins. The tubelet is built from the first frame, and if the corresponding pair of objects still exist in the following frames, the tubelet is extended. The tubelet can be extended until there is no paired detection object with the tubelet object in the next frame. The new tubelet can be initialized in any frame. For each pair of frames, if the paired objects do not belong to any existing tubelet, a new tubelet can be created.

The detection results are then optimized based on the linking tubelets. First, the bounding boxes are re-scored in the same tubelets, using good detection results to optimize the poor detection results. For the same object appearing in the adjacent frames, the predicted results should have the same class confidence no matter how large the shape change is. If the predicted object has low class confidence and belongs to a tubelet in which one other object has high class confidence, we could believe that the predicted object should have higher class confidence. So when re-scoring, the average classification confidence score of all detection objects in each tubelet is calculated and assigned to all objects in the tubelet, as shown in Equation (19), where N refers to the number of objects contained in a tubelet.

(19) $$score=\frac{1}{N}\sum_{i=1}^{N}{score}_{i}$$

Then, the coordinates of the bounding boxes are re-coordinated in the tubelets. Usually the movement of the object has a specific pattern and does not suddenly jitter, so the predicted bounding boxes in the adjacent frames usually change smoothly instead of changing drastically. We utilize this point to optimize the detected bounding boxes. Bounding box re-coordinating treats the four coordinates of the detection bounding boxes in a tubelet as time series with noise. A one-dimensional Gaussian filter is constructed along each time series, and the filter convolves a Gaussian function with each coordinate of the bounding boxes in the same tubelet. The sum of the convolution operation is used as the new coordinates of the detection objects in the tubelets, as shown in Equation (20).

(20) $$c_{i}=\sum_{i-j>f_{min}}^{i+j<f_{max}}c_{i+j}g\left(j\right)$$

g(·) represents the Gaussian function, as shown in Equation (21). The variable *x* represents the distance from the center point, while σ represents the standard deviation of the Gaussian distribution. *i* represents the current frame, $c_{i}$ represents the coordinate in the current frame, and $f_{max}$ and $f_{min}$ represent the starting and ending frames of the tubelet, respectively.

(21) $$g\left(x\right)=\frac{1}{\sqrt{2\pi \sigma }}e^{-\frac{x^{2}}{2\sigma^{2}}}$$

**Figure 6:**
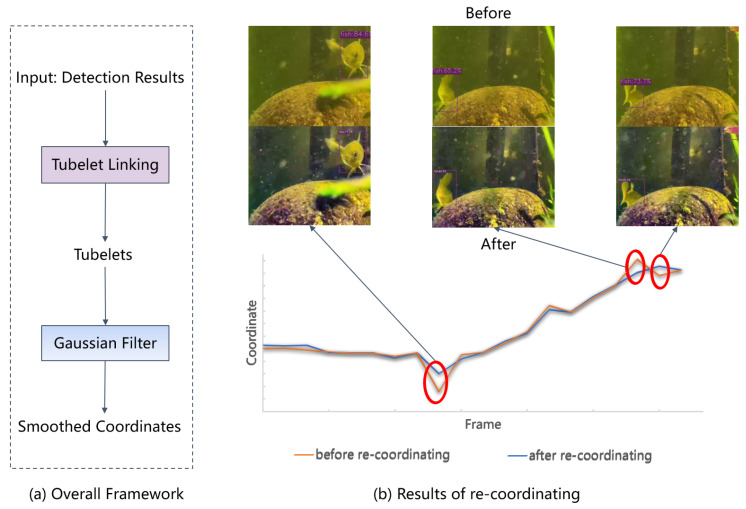
Results of re-coordinating. The red circle refers to the coordinates with significant differences before and after re-coordinating.

Re-coordinating applies a Gaussian filter to re-coordinate the coordinates of the same object in adjacent frames, and then use the new coordinates sequence as the bounding box coordinates of the detected objects in the tubelets. It reduces the bounding box jitters in video object detection. This effect can be seen in Figure 6. After re-coordinating, sudden changes of the abnormal coordinate values have been reduced and the overall variation of the bounding box coordinates of the same object becomes smoother as it suppresses the jitters.

## 4. Results

### 4.1. Experimental Environment Configuration

The experimental platform used in this paper is a GPU server with the Ubuntu operating system, with the following main hardware configuration: Intel(R) Xeon(R) Platinum 8338C CPU and RTX 3090 (24GB) GPU. The UWV-Yolox model is implemented using the deep learning framework Pytorch 1.10.1 and Python 3.8. In the experiments, the training input image size is set to 640 × 640, and the test input size is set to 576 × 576. The batch size is 16, and SGD [22] is used as the optimizer with a default weight decay of 0.0005, momentum of 0.9, and initial learning rate of 0.005. The NMS threshold is set to 0.5, and both Mosaic and Mixup probabilities are set to 1.

### 4.2. Dataset

The existing publicly available datasets are either image datasets or just a few numbers of videos, which are not suitable for training and testing the UWV-Yolox models in the paper. Considering that there is currently no universal publicly available underwater video dataset, we select suitable videos from the collected underwater video datasets to build a universal underwater video object detection dataset (UVODD).

By collecting and screening publicly available underwater datasets, three datasets with high data quality are selected, namely Brackish [23], UODD [24], and S-URPC2019 [25]. Brackish is an annotated ocean video dataset captured by cameras installed in temperate straits. The UODD dataset is established for underwater object detection tasks and contains data with multiple underwater scenes, multiple objects, large objects, and small objects. S-URPC2019 is a dataset for underwater object detection in the underwater robot target capture competition. We select parts of images in these datasets that can be converted into videos and add them to UVODD.

In terms of class consistency, considering the classes in publicly available underwater datasets, five common classes are selected as the UVODD dataset classes: echinus, holothurian, starfish, fish, and jellyfish. In terms of annotation format consistency, the ImageNet VID dataset [26] is an important dataset for evaluating video object detection models. Therefore, we use the storage format and annotation format of the ImageNet VID dataset as the standard for the UVODD dataset. All annotations in the UVODD dataset are converted into Pascal VOC format. Thus, the UVODD dataset is built, which can be used in various underwater video object detection tasks—not just limited to this paper. The UVODD dataset includes 74 videos, about 10,000 images, and over 20,000 annotated objects. It also covers different types of underwater scenes, a situation with multiple objects, and a situation with large and small objects. Thus the UVODD dataset has a certain degree of representativeness and diversity. The specific information of the UVODD dataset is shown in Table 1.

### 4.3. Experimental Results

#### 4.3.1. Evaluation Metrics

In order to objectively evaluate the performance of the model in underwater video object detection, we use the mAP@0.5 and FPS to validate. FPS refers to frames per second, which reflects the detection speed. mAP@0.5 refers to the mean average precision at an IOU threshold of 0.5, which is defined as follows:

(22) $$mAP=\frac{\sum_{i=1}^{n}{AP}_{i}}{n}$$

Here, *n* refers to the number of classes and AP refers to the average precision for a single class. Each class can calculate its precision and recall for an IOU threshold. Thus, the P-R curve can be obtained, and the AP value is the area under the curve, as shown in Equation (23).

(23) $$AP=\int_{0}^{1}P(r)dr$$

P(r) represents the precision when the recall is r. The precision represents the probability that the correctly predicted positive sample accounts for all predicted samples, as shown in Equation (24). The recall represents the probability of the correctly predicted positive samples among all positive samples, as shown in Equation (25).

(24) $$Precision=\frac{TP}{TP+FP}$$

(25) $$Recall=\frac{TP}{TP+FN}$$

*TP* represents the number of correctly detected positive samples, *FP* represents the number of incorrectly detected negative samples, and *FN* represents the number of incorrectly detected positive samples.

#### 4.3.2. Experimental Results with Different Data Augmentation Methods

**Figure 7:**
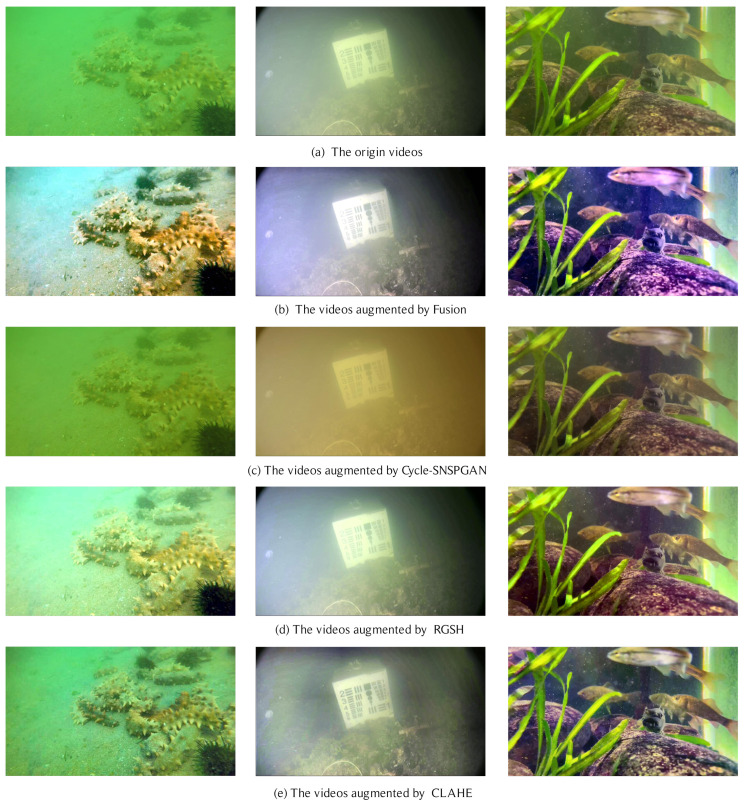
Results of video augmentation.

Before object detection, contrast enhancement processing with CLAHE is applied to the input videos. To verify the effect of the CLAHE, we conduct experiments to compare CLAHE with several data augmentation methods. Considering that there are no paired original images and augmented images in the dataset, methods with supervised learning are not possible. Therefore, we select traditional data augmentation methods and GAN-based data augmentation methods for experiments. Figure 7 shows the augmented videos of different data augmentation methods. The fusion augmentation method makes the videos clearer but causes significant color deviation for the objects in the videos. The Cycle-SNSPGAN method does not have an obvious dehazing effect on the videos. The RGSH method corrects the contrast and color of the videos but some parts are excessively enhanced, causing an exposure phenomenon. Overall, the CLAHE method is the best augmentation method for underwater videos.

Table 2 shows the detection results of the detection model when different augmentation methods are applied to it on the UVODD dataset. It shows that the CLAHE method improves the mAP@0.5 of underwater video object detection by 1.2% compared with the result without augmentation, while other data augmentation methods do not significantly improve or even weaken the detection performance. It indicates the effectiveness of using the CLAHE contrast enhancement method to augment underwater videos.

#### 4.3.3. Experimental Results with Individual Improvement

This paper proposes the UWV-Yolox model with five improvements. To verify the meaningfulness of each improvement and its ability to improve the performance of underwater video object detection, we conduct the experiments. Each improvement is individually added to the original UWV-Yolox model, and we then train and test the model on the UVODD dataset. The results are shown in Table 3.

It can be seen that each improvement improves the performance of the UWV-Yolox model, indicating their effectiveness in improving the model’s ability to detect objects in underwater videos. Among all, the jitter loss has a particularly significant effect on detection. Jitter is a common phenomenon in video object detection, and weakening jitters through jitter loss is beneficial for improving video detection performance. The CLAHE method applied to enhance contrast in input videos and the frame-level optimization module applied to optimize detection results increase the detection mAP@0.5 by at least 1.2%. The addition of a CA mechanism to the model’s backbone also improves the model’s ability to detect objects, increasing the detection mAP@0.5 by 0.7%. The replacement of regression loss is not significantly improving the performance, as it is only 0.2% higher than the baseline. It indicates that the overlapping area plays a major role in evaluating the proximity between predicted and ground truth boxes.

#### 4.3.4. Results of the Ablation Experiments

We conduct ablation experiments to explore the effectiveness of augmenting videos using CLAHE, adding a CA mechanism, adding jitter loss, replacing regression loss, and adding a frame-level optimization module in the UWV-Yolox model. These improvements are tested separately in six models trained and tested on the same UVODD dataset, as shown in Table 4.

From Table 4, it can be seen that each improvement in the UWV-Yolox model improves the detection results. Considering that the input videos interfered with by the underwater environment are blurry and of low contrast, the underwater videos are first processed with CLAHE to enhance the contrast. It makes mAP@0.5 improved by 1.2%, indicating that enhancing the contrast improves the clarity of the object features in the videos. Next, the CA module is added to the feature extraction backbone network to augment the representations of the objects of interest, and the mAP@0.5 is 0.5% higher than when only augmenting the videos. Additionally, since jitters are inevitable during the detection of bounding boxes in videos, the jitter loss is added to the loss function, improving by 0.5% for detection. It indicates that jitters are a common occurrence in videos and an important optimization point for video detection. Subsequently, the IOU regression loss is replaced with $CIOU_{log}$ regression loss, which considers the relationship between the overlapping area, position, and shape of the predicted and ground truth boxes. It improves the proximity between the predicted and ground truth boxes and is 0.8% better than the former experiment. Finally, since the objects in adjacent frames of a video are usually similar, a frame-level optimization module is added to the model. It constructs tubelets between adjacent frames for bounding box re-scoring and re-coordinating, improving by 0.2% in underwater video detection performance.

We compare the experimental results of adding each improvement individually and the ablation experiments. When comparing the result of directly adding jitter loss with adding jitter loss after data augmentation and adding the CA module, we found that the improvement of mAP@0.5 decreased from 2.4% to 0.5%. This indicates that data augmentation and the CA module lead to improvements in underwater video object detection and reduce the effectiveness of the jitter phenomenon as well. The same as the frame-level optimization. However, replacing the regression loss improved the detection mAP@0.5 from 0.2% in the separation experiment to 0.8% in the ablation experiment. It suggests that, after reducing the effectiveness of jitter, the overlapping area is not sufficient to evaluate the proximity between the predicted and ground truth boxes, so the influence of position and shape is also significant.

#### 4.3.5. Experimental Results of Different Model

To verify the effectiveness of the proposed UWV-Yolox model in underwater video object detection, we conduct some comparative experiments to compare the UWV-Yolox model with other detection models on the UVODD dataset. The results are shown in Table 5.

From the table, it can be seen that the mAP@0.5 of the UWV-Yolox model reaches 89.0%, the highest among all models, which indicates that the model has the best detection performance for underwater videos. Compared to the TransVOD_Lite video detection model, the UWV-Yolox model has higher mAP@0.5 and faster speed. This indicates that the UWV-Yolox model performs better in detecting underwater videos with low contrast and blurriness. As for the Boosting R-CNN, an underwater detection model, the UWV-Yolox model increases the mAP@0.5 by 11.6% and fastens the detection speed. It shows that the UWV-Yolox model performs better for underwater scenes and makes full use of the video information. The UWV Yolox model improves the mAP@0.5 by 6.7% compared to the Yolov5 model but slows down the detection speed. The process of augmenting underwater videos and the optimization of detection results using frame relationships in the UWV-Yolox model benefit for detecting objects in the underwater video but also consume more detection time, which is not conducive to real-time detection. The YOLOV model learns the spatiotemporal relationships in videos during model training, but its generalization ability for underwater scenes is weak. The UWV-Yolox model not only utilizes video information but also improves the detection of underwater scenes, so it increases the mAP@0.5 by 4%, requiring more detection time. Compared to the Yolox model, the UWV-Yolox model improves the mAP@0.5 by 3.2% with close detection speed. While ensuring detection speed, It improves the detection performance for underwater videos without slowing down the detection speed.

**Figure 8:**
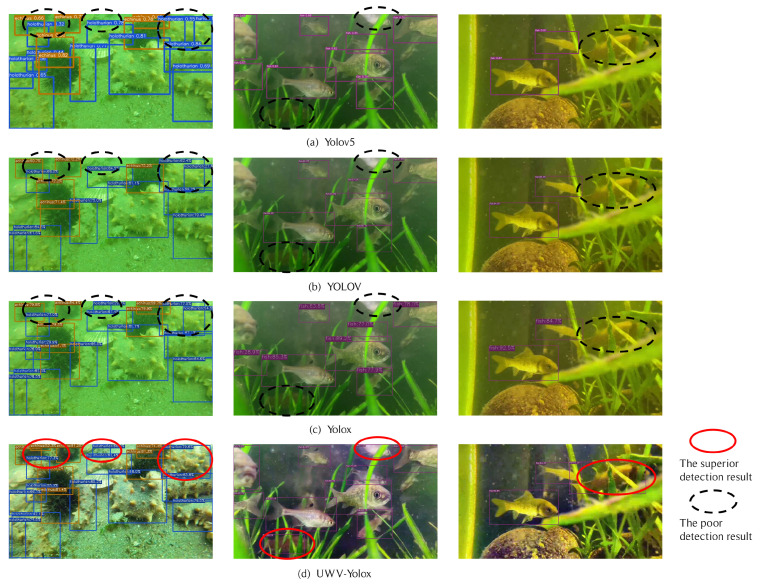
The detection results of different underwater video object detection models.

Figure 8 shows the detection results of various detection models for underwater video object detection. The red ellipses highlight the superior performance of the UWV-Yolox model compared with the detection results with the black ellipses. It can be found that most of the confidence of objects detected by the UWV-Yolox model is higher than the confidence detected by other models. A few objects show a slight decrease in confidence due to frame-level optimization, which uses high-confidence objects to optimize the same object with low confidence in the adjacent frames. Moreover, the UWV-Yolox model can detect objects that cannot be detected by other models, such as partially occluded objects, incompletely shaped objects, and blurry objects. This indicates that augmenting the underwater video and integrating the CA module to utilize the position relationship of blurry objects in the UWV-Yolox model is beneficial for improving performance in underwater videos. Compared to other models, the UWV-Yolox model also reduces wrong object detection, such as detecting one object as multiple objects. In addition, the predicted bounding boxes of the UWV-Yolox model more accurately locate the objects, and the changes of bounding boxes between adjacent frames are smoother, suppressing the jitters.

Overall, the UWV-Yolox model improves the performance of object detection in underwater videos. It improves the classification confidence of most objects. It also enhances the detection ability for occluded, incomplete, and blurry objects. Furthermore, the UWV-Yolox model makes the predicted bounding boxes closer to the ground truth bounding boxes and smoothens the changes of the bounding boxes between adjacent frames, suppressing the jitters. Although the detection speed of the UWV-Yolox model is not fast, it has reached the level of real-time detection. However, the frame-level optimization module reduces the confidence of some high-confidence objects by averaging the confidence of the same tubelets to optimize the low-confidence objects. Additionally, the augmentation of underwater video and the frame-level optimization module slow down the detection speed.

## 5. Conclusions

We propose the UWV-Yolox model to address the issue of underwater video object detection. In the model, we use the Contrast Limited Adaptive Histogram Equalization method to enhance video contrast. In the backbone for feature extraction, we propose a new CSP_CA module by adding a Coordinate Attention module to the CSP module. As for the loss function, we add jitter loss to optimize the prediction of object detection boxes in the design of the loss function. Frame-level optimization module is also added to optimize the detection results. By conducting experiments, we validate the effectiveness of the proposed UWV-Yolox model. On the UVODD dataset built in the paper, the mAP@0.5 of the UWV-Yolox model reaches 89.0%, which is 3.2% better than the original Yolox model.

UWV-Yolox provides a new perspective for solving the task of underwater video object detection, and the improvements of the UWV-Yolox can be flexibly applied to other models to improve the detection results. The UWV-Yolox model will soon also be applied to an eco-environment research program for the Environmental Protection Agency to explore underwater biodiversity and environmental stability in rivers.

In the future, we plan to continue cooperating with the Environmental Protection Agency to obtain more underwater video data to increase the dataset required for training. Furthermore, future research will make more in-depth lightweight improvements on UWV-Yolox, thus fastening the detection speed and reducing the demand for hardware configuration to expand the application scenarios of the model.

## Figures and Tables

**Table 1:** UVODD dataset.

	The Number of Videos	The Number of Images	The Number of Objects
Train	59	6886	15,141
Test	15	1773	5930
Sum	74	8659	21,071

**Table 2:** Results with different augmentation methods.

Model	Augmentation Methods	mAP@0.5(%)
Yolox	None	85.8
Improved Yolox	Fusion [27]	81.4 (−4.4)
Improved Yolox	Cycle-SNSPGAN [28]	82.9 (−2.9)
Improved Yolox	RGSH	86.0 (+0.2)
**UWV-Yolox**	**CLAHE**	**87.0 (+1.2)**

Red color means the increase of mAP@0.5. Green color means the decrease of mAP@0.5. Bold means the results of the UWV-Yolox model. The contents in the ( ) mean the mAP@0.5 value compared with the value without the augmentation method.

**Table 3:** Results of the original UWV-Yolox model with individual improvement.

Methods	mAP@0.5(%)	Parameters
baseline	85.8	99.00 M
+ Regression loss	86.0 (+0.2)	99.00 M
+ Coordinate Attention	86.5 (+0.7)	82.66 M
+ CLAHE	87.0 (+1.2)	99.00 M
+ Frame-level optimization	87.3 (+1.5)	99.00 M
+ Jitter loss	88.2 (+2.4)	99.00 M

Red color means the increase of mAP@0.5. The contents in the ( ) mean the mAP@0.5 value compared with the value of baseline.

**Table 4:** Results of the ablation experiments.

CLAHE	CA Module	Jitter Loss	Regression Loss	Frame-Level Optimization	mAP@0.5(%)
					85.8
✓					87.0 (+1.2)
✓	✓				87.5 (+0.5)
✓	✓	✓			88.0 (+0.5)
✓	✓	✓	✓		88.8 (+0.8)
✓	✓	✓	✓	✓	89.0 (+0.2)

Red color means the increase of mAP@0.5. The contents in the ( ) mean the mAP@0.5 value compared with the upper value.

**Table 5:** Results of different detection models.

Model	Backbone	Input Size	Batch Size	mAP@0.5(%)	FPS
TransVOD_Lite [29]	ResNet-101	600 × 600	4	69.0	14.9
Boosting R-CNN [30]	ResNet-50	1333 × 800	4	77.4	25.4
Yolov5	CSPDarknet	640 × 640	32	82.3	156.2
YOLOV [31]	CSPDarknet	640 × 640	32	85.0	104.1
Yolox	CSPDarknet	640 × 640	16	85.8	75.6
**UWV-Yolox**	**CA_CSPDarknet**	**640 × 640**	**16**	**89.0**	**71.8**

Bold means the results of the UWV-Yolox model.

## Data Availability

Not applicable.
